# Acceptance of telemedicine among care personnel in inpatient and outpatient elderly care: a systematic review

**DOI:** 10.1186/s12877-025-06786-9

**Published:** 2025-11-22

**Authors:** Birthe Aufenberg, Julia Offermann, Sophie Pauge, Martina Ziefle, Wolfgang Greiner

**Affiliations:** 1 Department of Health Economics and Health Care Management, School of Public Health, Bielefeld University, Bielefeld, Germany; 2 Human-Computer Interaction Center (HCIC), RWTH Aachen University, Aachen, Germany

**Keywords:** Telemedicine, Acceptance, Perceptions, Elderly care

## Abstract

**Background:**

Healthcare systems face substantial challenges due to the growing number of elderly people in need of care and the shortage of nursing staff. As a result, it is difficult to provide timely and appropriate healthcare. One potential solution could be the use of telemedicine. However, care personnel’s acceptance of the use of telemedicine is often a challenge. The aim of this systematic review is to determine the status quo of acceptance of telemedicine, provide an overview of motivators and barriers associated with the use of telemedicine, and identify factors predicting acceptance of telemedicine among care personnel working in elderly care.

**Methods:**

An electronic search was conducted in MEDLINE, PsycInfo, and Embase, supplemented by a hand search. Articles were identified based on predefined inclusion and exclusion criteria. Study characteristics and relevant results were extracted. The methodological quality of the included studies was assessed using the *Quality Assessment with Diverse Studies* tool.

**Results:**

A total of 16 studies were considered eligible for inclusion. Overall, the included studies highlighted positive attitudes of care personnel towards telemedical applications in inpatient and outpatient elderly care, but also showed some critical views and ambivalent opinions about these types of care. As part of that, several motivators and barriers concerning telemedicine were identified. Motivators and barriers referred to general, organizational, personnel-related, patient-related, family-related, and technology-related aspects. Notably, there were more motivators than barriers in all included studies taken together. Few studies investigated factors influencing care personnel’s acceptance of telemedicine in depth, although there is some evidence that demographic characteristics impact the acceptance of telemedical applications.

**Conclusions:**

The results of this systematic review indicate that the acceptance of telemedicine among care personnel is positive overall. A range of motivators has been identified, highlighting the perceived potential of telemedicine to meet current challenges in elderly care. However, some challenges remain. In order to realize the full potential of telemedicine in elderly care, it is necessary to address the identified barriers.

**Supplementary Information:**

The online version contains supplementary material available at 10.1186/s12877-025-06786-9.

## Background

Worldwide, the demand for nursing and medical care of geriatric patients is constantly increasing due to the demographic change and the rise in chronic diseases, multimorbidity, and care dependency [1, 2]. At the same time, increasing workloads and staff shortages in the healthcare sector make it more difficult to provide timely, needs-based, and high-quality primary and acute care to geriatric patients in nursing homes and at home [3–7]. In nursing homes, geriatric patients represent the most vulnerable group of individuals, given their increased susceptibility to illness and the prevalence of multiple chronic conditions [8–10]. Consequently, they require a considerable level of acute medical care. In this context, particular attention should be given to the organization of acute medical emergency care for geriatric patients, as these situations often lead to unnecessary hospitalizations, especially outside general practitioners’ office hours [11]. Such hospitalizations are associated with an increased risk of health deterioration and impose a substantial financial burden on the healthcare system [12].

The use of telemedicine could be one way to address these issues and could support the delivery of care to geriatric patients. Telemedicine is broadly defined as “the use of electronic information and communications technologies to provide and support healthcare when distance separates the participants” [13]. In this article, telemedicine is understood as patient-centered healthcare services delivered with the involvement of healthcare providers using information and communications technologies [14, 15]. It is often applied in form of video consultations between a patient and care personnel on the one side and medical personnel on the other, a method that was used extensively during the Covid-19-pandemic [16–18]. Telemedicine and other digital health technologies promise advances in healthcare delivery. For instance, the ability to remotely connect with health care professionals improves geriatric patients’ access to (specialized) healthcare regardless of geographic proximity and reduces costs to the healthcare system [19–21]. Other potential benefits include improvements in the quality and efficiency of healthcare services, particularly through continuity of care and enhanced interprofessional communication [22].

However, pilot projects in telemedicine often face challenges in sustaining the use of telemedical applications beyond the project phase, preventing them from becoming fully established in regular care [23, 24]. This is due to barriers such as limited time and financial resources, inadequate infrastructure and equipment, insufficient skills, preference for in-person consultations, and ethical concerns [25–27]. In addition, stakeholder acceptance of telemedicine remains often a challenge. Studies have found that, in particular, care personnel are skeptical about the use of assistive technology in older age [28, 29]. This skepticism is also reflected in qualitative research on nurses’ experience with telecare, which highlights relational and professional concerns regarding the use of digital technologies in care [30]. Yet, telemedicine acceptance is crucial for the successful adoption and diffusion of telemedical applications. Diverse models and approaches are used to examine technology acceptance, focusing not only on usability or technological features as important components of acceptance, but also on support and influence from colleagues, organizational conditions and other factors [e.g., 31, 32]: These investigations are predominantly based on classical technology acceptance models such as Davis’ *Technology Acceptance Model* (TAM) [33] and Venkatesh’s *Unified theory of acceptance and use of technology* (UTAUT) [34]. While general technology acceptance models such as TAM and UTAUT provide foundational insights into factors influencing technology adoption, their application in nursing and elderly care necessitates consideration of the unique professional, ethical, and relational dimensions inherent in these settings. TAM emphasizes perceived ease of use and perceived usefulness [33], while UTAUT introduces constructs like performance expectancy, effort expectancy, social influence, and facilitating conditions [34]. However, both neglect the complex interplay between technology and the socio-organizational context in which healthcare professionals operate. To address this, nursing- and healthcare-specific frameworks have been developed. Socio-technical models and considerations [e.g., 35, 36], for instance, emphasize the interaction between people, technology, and organizational structures, highlighting that successful technology adoption depends not only on individual attitudes but also on integration into workflows and organizational practices. These models advocate for a holistic approach to technology implementation, considering both technical and social factors. Similarly, the Normalization Process Theory (NPT) offers a framework for understanding how new technologies become routinely embedded in healthcare practices [37]. The NPT identifies mechanisms such as coherence, cognitive participation, collective action, and reflexive monitoring that influence the integration of innovations into everyday work.

Integrating these models into the study of telemedicine acceptance allows for a nuanced understanding of how factors like professional identity, ethical considerations, and relational dynamics affect technology adoption. For example, nurses’ professional values and the nature of patient relationships can significantly influence their acceptance of telemedicine solutions. Therefore, expanding the theoretical framework to include these perspectives is essential for developing strategies that promote the successful integration of telemedicine in nursing and elderly care.

As acceptance is essential for the successful implementation of telemedicine in the long term, it is necessary to evaluate the stakeholders’ acceptance of a telemedical application holistically during the implementation phase [38]. As care personnel plays an important role in the healthcare process and mediates between patients and medical personnel, this occupational group should be given particular attention. While some systematic reviews already provide a comprehensive insight into the status quo of acceptance and the reasons for (non-)use of telemedicine [39–41], the perspective of care personnel is not consistently considered.

Therefore, this systematic review aimed to (1) determine the status quo of telemedicine acceptance, (2) provide an overview of motivators and barriers as facilitators for the acceptance and adoption of telemedicine, and (3) identify factors predicting telemedicine acceptance of care personnel working in inpatient and outpatient elderly care.

## Methods

This systematic review was conducted in accordance with the *Preferred Reporting Items for Systematic Reviews and Meta-Analyses (PRISMA)* statement [42].

### Eligibility criteria

According to the eligibility criteria (see Table 1), studies were included if they focused on care personnel in inpatient and/or outpatient care of the elderly. This applied as well to studies examining the general acceptance of healthcare workers, including care personnel. Interventions involving telemedicine were considered when the healthcare service was patient-centered and care personnel was involved in its delivery. The outcome of interest was the acceptance of the respective telemedical intervention by the care personnel. Moreover, studies with a qualitative and quantitative design that were either published in English or German were considered. As the acceptance of telemedicine has the potential to change over time (e.g., [56]), only studies published after the year 2000 were considered.

**Table 1:** Eligibility criteria of the systematic review.

Criteria	Inclusion	Exclusion
Population	Care personnel in inpatient and outpatient elderly care	Healthcare professionals in general excluding care personnel
Intervention	Patient-centered healthcare service delivered through a telemedical technology with the involvement of care personnel (e.g. teleconsultation), including hypothetical or scenario-based interventions	Digital healthcare services without focus on telemedicine (e.g. electronic management of patient data, self-monitoring)
Outcomes	Acceptance of telemedical technology	All other outcomes
Study design	Quantitative studies (intervention studies, observational studies), qualitative studies	Comments, editorials, letters, case studies/reports, narrative reviews, systematic reviews, and meta analyses
Publication language	English, German	All other languages
Publication date	Publication after 2000	Publication before 2000

### Information sources, search strategy, and study selection

The electronic search was conducted in three databases: MEDLINE (via PubMed), PsycInfo (via EBSCO), and Embase (via Elsevier). In addition, further studies were identified through hand search and cross-checking of references in the included studies. The search strategy consisted of four parts (see Appendix 1), with different search terms and keywords used for each part. Depending on the database, specific keywords, such as MeSH-Terms and Emtree-Words were used, as well as truncations. A restriction to title/abstract was made. The four parts of the search strategy covered acceptance, telemedicine, nursing staff, and care setting, as these reflect the main aims of our study.

The study selection was performed in two steps based on the eligibility criteria. First, the titles and abstracts of the identified studies were checked for relevance. Potentially suitable studies were then screened in full text. Reasons for the exclusion of full text studies were documented. Study selection was carried out independently by two reviewers (SP, BA); a third reviewer (JO) was consulted in case of disagreement. This procedure was also implemented for quality assessment and data extraction.

### Data extraction and qualitative synthesis

The results of the included studies were reviewed and information on each study was extracted. In addition to study characteristics, the telemedical intervention, the definition of acceptance and its measurement, and results were extracted. The results were categorized by status quo of acceptance, motivators and barriers, and predicting factors of telemedicine acceptance, following the objectives of the study.

Data from the extracted results on motivators and barriers in the included studies were synthesized qualitatively, as the study design and study populations were relatively open. (Sub)categories for both aspects were developed inductively and the extracted results were assigned to these (sub)categories. All categorizations were cross-checked and, if necessary, discussed within the team of authors.

### Quality appraisal

To determine the validity and quality of the included studies, the risk of bias was assessed. As the eligibility criteria foresaw the inclusion of studies with a quantitative and/or a qualitative methodology, the *Quality Assessment with Diverse Studies* (QuADS) tool [43], which takes into account both research methods, was used. The QuADS tool consists of 13 criteria, each measured on a four-point Likert scale, with three being the best. Two authors (SP, BA) independently appraised the quality based on the information provided in the selected articles following the QuADS User Guide v1.0.

## Results

**Fig. 1:**
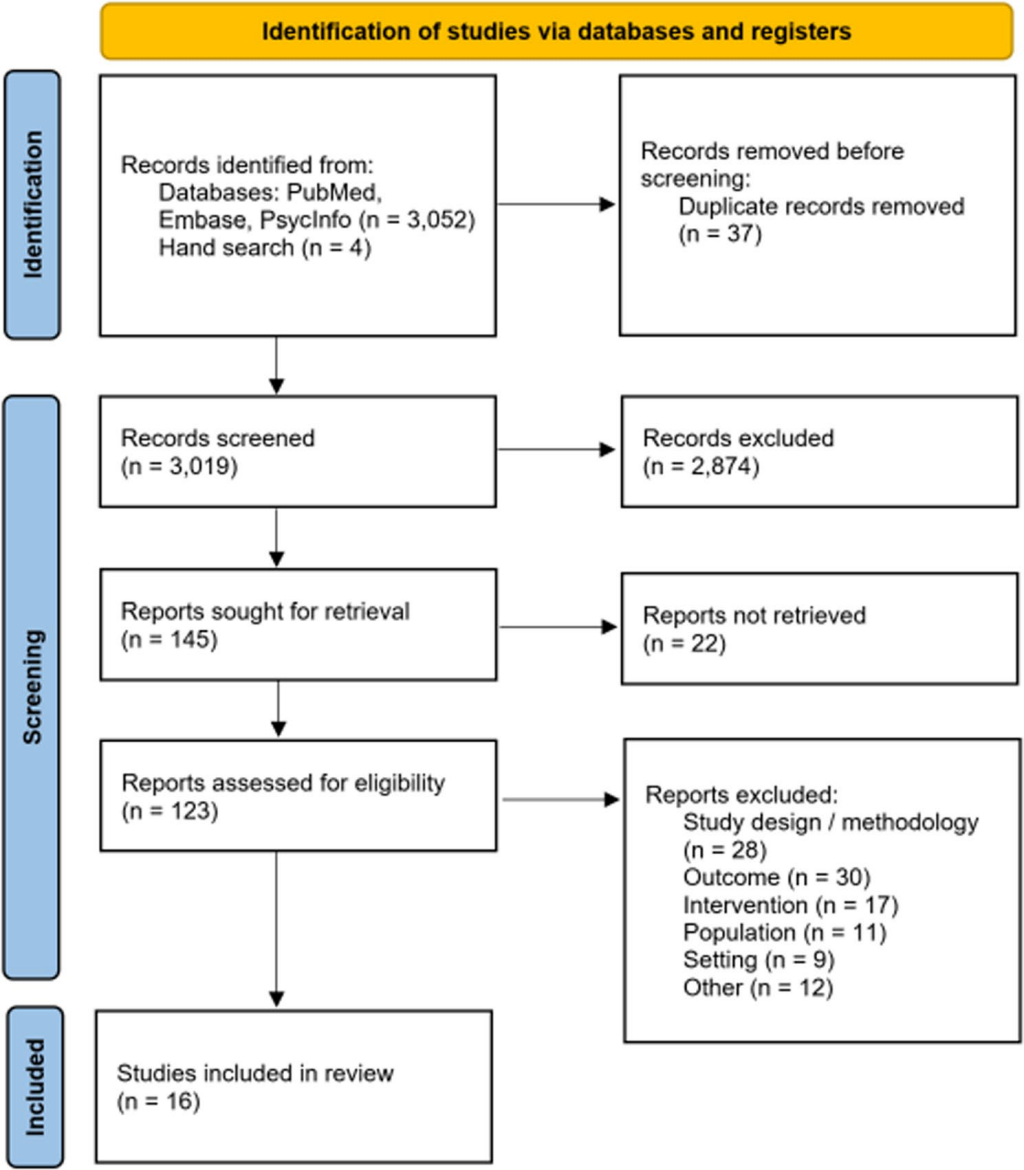
PRISMA flow chart of study selection [42]

The search resulted in 3,052 potentially eligible studies up until January 2022 and was supplemented by four additional articles identified through hand search. After duplicates were removed and title/abstracts were screened, 123 full texts were assessed for eligibility. A total of 16 studies were included in the systematic review (see Fig. 1).

### Study characteristics

A comprehensive overview of the characteristics of the 16 included studies is given in Table 2. The majority of the studies were conducted in Europe [44, 46, 49, 51–55, 57]. Almost half of the studies used a qualitative study design [45, 47, 52–55, 57], while six studies employed a quantitative study design using questionnaires and surveys instead, that were either self-developed [48–50, 58, 59] or a validated tool [60], and three studies combined both methods [44, 49, 51]. Half of the studies examined the acceptance of telemedicine in inpatient elderly care [44, 47, 49–53, 57] and seven studies were conducted in outpatient elderly care [45, 46, 48, 55, 58–60]. Only one study focused on the transition phase from hospital to municipality care [54]. Most of the studies investigated the acceptance of an actually employed telemedical application [44, 47, 48, 51–54, 57–60]. In contrast, five studies examined the acceptance on a hypothetical level [45, 46, 49, 50, 55].

**Table 2:** Study characteristics of the included studies

Study	Country	Observation period	Study approach	Population	Setting	Intervention	Acceptance	
							Operationalization	Measurement
Appleman et al. [48]	USA	-	Quantitative	Care personnel, medical personnel, administrative and support personnel (*n* = 5)	Outpatient elderly care	Video consultation between care and support personnel in the patients’ home and medical personnel at a hospital (*n* = 11 visits with 9 patients)	Satisfaction with video consultations	Questionnaire (self-developed)
Chang et al. [50]	Taiwan	04/2007	Quantitative	Care personnel (*n* = 79)	Inpatient elderly care	Scenario-based: telemedicine defined as interactive audio and video telecommunications, telemonitoring and alarming systems and electronic, Internet-based connection between hospital and nursing home	Attitudes, knowledge and perspectives, concerns and worries, expectations of benefits related to telemedicine	Questionnaire (self-developed)
Cormi et al. [44]	France	Quantitative assessment: 04/2018–04/2021 Qualitative assessment: 09/2020–11/2020	Mixed-methods	Care personnel, medical personnel, management and leadership personnel (*n* = 11 in 26 nursing homes)	Inpatient elderly care	Synchronous video teleconsultation program conducted between nursing homes and a general hospital (*n* = 590 teleconsultations)	Quantitative part: teleconsultation activity; qualitative part: motivations to conduct and experiences with teleconsultations	Quantitative part: descriptive analysis of teleconsultation activity; qualitative part: semi-structured interviews
DelliFraine et al. [58]	USA	2004–2005	Quantitative	Care personnel (*n* = 884 in 29 mobile nursing services)	Outpatient elderly care	Telemedicine program used for data collection, interactive visits, communication with patients, medical personnel and others	Usage of telemedicine, perceived efficiency and effectiveness of telemedicine	Questionnaire (self-developed)
Eastman et al. [59]	Australia	04/2020–05/2020	Quantitative	Care personnel (*n* = 22)	Outpatient elderly care	Telemedicine consultation between a patient and care personnel using a telecommunication device	Interactions with and opinions of telemedicine consultations	Questionnaire (self-developed)
Kozikowski et al. [45]	USA	02/2017–08/2017	Qualitative	Care personnel, medical personnel, personnel in the social sector, administrative and support personnel (interviews – *n* = 16; focus groups – *n* = 20)	Outpatient elderly care	Scenario-based: telemedicine defined as the remote provision of health care through various telecommunication technologies	Perspectives and motivations regarding the adoption of telemedicine technology to scale health care provision	Semi-structured interviews, focus groups (*n* = 3)
Lemon et al. [60]	Australia	t0 = 10/2013; t1 = 04/2015	Quantitative	Care personnel (*n* = 10)	Outpatient elderly care	Telemedicine system that allows the storage of patient data and the conduct of video consultations among care and medical personnel	Perceptions of a telemedicine system	Questionnaire (validated tool: System Usability Scale)
Newbould et al. [57]	United Kingdom	-	Qualitative	Care personnel, administrative and support personnel, management and leadership personnel (*n* = 19)	Inpatient elderly care	Videoconferencing service for care personnel seeking support from medical personnel for the care of residents	Factors affecting the uptake and sustainability of videoconferencing	Guideline-based interviews
O’Sullivan et al. [49]	Germany	2015	Mixed-methods	Care personnel, administrative and support personnel (*n* = 216 in 10 nursing homes)	Inpatient elderly care	Scenario-based: telemedicine defined as ICT with features that allow interactive communication and multimedia functions	Beliefs associated with the intention to use, usage behavior, attitudes and needs regarding telemedicine in nursing homes	Quantitative part: survey (self-developed, integration of a validated tool: TA-EG questionnaire, *n* = 205); qualitative part: semi-structured stakeholder interviews (*n* = 11)
Øyen et al. [46]	Norway	01/2014–05/2014	Quantitative	Care personnel, management and leadership personnel (*n* = 155)	Outpatient elderly care	Scenario-based: telemedicine defined as ICT with features that allow online communication, patient care, treatment or supervision	Attitudes to use telemedicine in home care nursing	Questionnaire (self-developed)
Piau et al. [51]	France	t0 = 2015; t1 = 2017	Mixed-methods	Care personnel, medical personnel (in 10 nursing homes)	Inpatient elderly care	Telemedicine consultation for the management of neuropsychiatric symptoms of dementia patients (*n* = 180 teleconsultations)	Perception of telemedicine	Quantitative part: semi-structured interviews (t0, t1); qualitative part: questionnaire (self-developed, t1)
Plunger et al. [52]	Austria	07/2020–08/2020	Qualitative	Care personnel, medical personnel, management and leadership personnel (*n* = 14 in 7 nursing homes)	Inpatient elderly care	Tablet- and videoconferecing-based telemedicine application used for virtual communication between medical, care personnel and residents during virtual medical visits	Technology readiness, acceptance, usability, experiences	Semi-structured interviews
Sävenstedt et al. [53]	Sweden	11/1999–02/2000	Qualitative	Care personnel, medical personnel (*n* = 6)	Inpatient elderly care	Videoconference between care and medical personnel to replace traditional ward rounds (*n* = 15)	interactions between nurses and the doctor; experiences with teleconsultations	Videorecording of teleconsultations; open interviews
Silsand et al. [54]	Norway	05/2020–06/2020	Qualitative	Care personnel, medical personnel, administrative and support personnel, management and leadership personnel (*n* = 19)	Transition between inpatient and outpatient elderly care	Videoconference between patient-centered team members and other collaborators (GPs, hospital wards, mobile nursing services)	Experience of using videoconferencing	Semi-structured focus groups (*n* = 4)
Toh et al. [47]	Singapore	02/2014-07/2014	Qualitative	Care personnel, medical personnel, administrative and support personnel, management and leadership personnel (*n* = 24 in 3 nursing homes)	Inpatient elderly care	Real-time telemedicine consultation with medical personnel	Perceptions of and experience with telemedicine system	Focus groups (*n* = 7), semi-structured interviews (*n* = 2)
van der Cingel et al. [55]	Netherlands	09/2016–07/2017	Qualitative	Care personnel (*n* = 43)	Outpatient elderly care	Scenario-based: telemedicine defined as care-interventions for patients in which (digital) technology is used	Assessment of electronic Health interventions	Think aloud interviews, focus group (*n* = 1)

*GPs* General practitioners, *ICT* Information and Communication Technologies, *USA* United States of America

### Methodological quality of the included studies

Based on the assessment, the included quantitative and qualitative studies were mainly of moderate study quality (see Table 3). Overall, the studies adequately defined the study objectives and populations. While the study designs and data collection instruments were mostly adequate to measure the telemedicine acceptance of the healthcare professionals, the description of the data collection process was not exhaustively captured. However, the greatest risk of bias, occurred due to missing underlying theories that framed the studies, with the exception of two studies [45, 60]. Furthermore, most of the studies did not involve relevant stakeholders in the research design.

**Table 3:** Methodological quality assessment of the included studies using the quality assessment for diverse studies [41]

QuADS Criteria	Appleman et al. [44]	Chang et al. [45]	Cormi et al. [46]	DelliFraine et al. [47]	Eastman et al. [48]	Kozikoswki et al. [49]	Lemon et al. [50]	Newbould et al. [51]	O’Sullivan et al. [52]	Øyen et al. [53]	Piau et al. [54]	Plunger et al. [55]	Sävenstedt et al. [43]	Silsand et al. [56]	Toh et al. [57]	van der Cingel et al. [42]
1. Theoretical or conceptual underpinning to the research	0	0	0	1	0	3	0	0	3	1	0	0	0	0	0	0
2. Statement of research aim/s	2	3	3	3	3	3	3	3	3	3	2	2	3	3	3	3
3. Clear description of research setting and target population	2	3	3	2	2	3	3	2	3	3	2	3	3	3	3	3
4. The study design is appropriate to address the stated research aim/s	2	2	3	2	2	2	2	2	3	2	3	2	3	2	2	2
5. Appropriate sampling to address the research aim/s	0	2	0	0	0	2	2	3	2	2	2	2	1	2	2	1
6. Rationale for choice of data collection tool/s	0	1	0	0	0	2	2	3	3	3	1	2	2	0	1	3
7. The format and content of data collection tool is appropriate to address the stated research aim/s	2	2	2	2	2	2	2	2	3	3	3	2	2	2	2	3
8. Description of data collection procedure	2	0	1	2	2	2	3	0	3	3	2	2	2	2	2	3
9. Recruitment data provided	1	2	1	2	0	2	3	2	3	2	0	3	3	2	0	0
10. Justification for analytic method selected	0	0	1	0	0	1	0	2	3	2	1	3	3	1	2	2
11. The method for analysis was appropriate to answer the research aim/s	2	2	2	2	2	2	2	3	3	3	2	3	2	2	2	2
12. Evidence that the research stakeholders have been considered in research design or conduct	0	0	0	0	1	1	0	2	0	1	2	1	1	0	0	0
13. Strengths and limitations critically discussed	0	3	2	2	2	2	2	2	2	2	3	2	0	2	1	1

Scoring guideline: 0 = not at all satisfied; 1 = very slightly satisfied; 2 = moderately satisfied; 3 = completely satisfied

*QuADS* Quality Assessment for Diverse Studies

In general, the quantitative studies tended to be rated slightly lower in quality than the qualitative studies. Of all the studies, one quantitative study [46] and one mixed-methods study [49] received the highest scores. Although the study methods chosen in the quantitative studies generally seemed appropriate to answer the research questions, a justification of them was more often lacking than in the qualitative studies. A better reflection on one’s own limitations would have improved the overall quality rating, especially in the qualitative studies.

### Status quo of acceptance

Overall, the acceptance of using telemedicine in elderly care was investigated in all analyzed studies taking the *perception* of telemedicine, previous *experiences* with telemedicine as well as *interest* in and *satisfaction* with telemedical applications into account. Starting with the *perception* of telemedical applications, telemedicine was positively perceived in most of the studies, and its potential in healthcare supply was acknowledged. Partly, also critical views of care personnel on telemedicine were described [52, 54, 58]. Focusing on concrete *experiences* with telemedical applications, three studies [44, 48, 53] reported exact numbers of conducted telemedical consultations. Two studies mentioned that nurses rarely use digital technologies in care in general [49, 58] which underlines the low baseline experience and helps to contextualize the acceptance of telemedical applications. Beyond that, four studies highlighted satisfaction with telemedical visits and reported only positive evaluations [48, 50, 59, 60]. *Interest* in using telemedicine was explicitly described in two studies [49, 50], reporting that the majority was interested in and aware of telemedicine.

### Motivators of telemedicine use

**Fig. 2:**
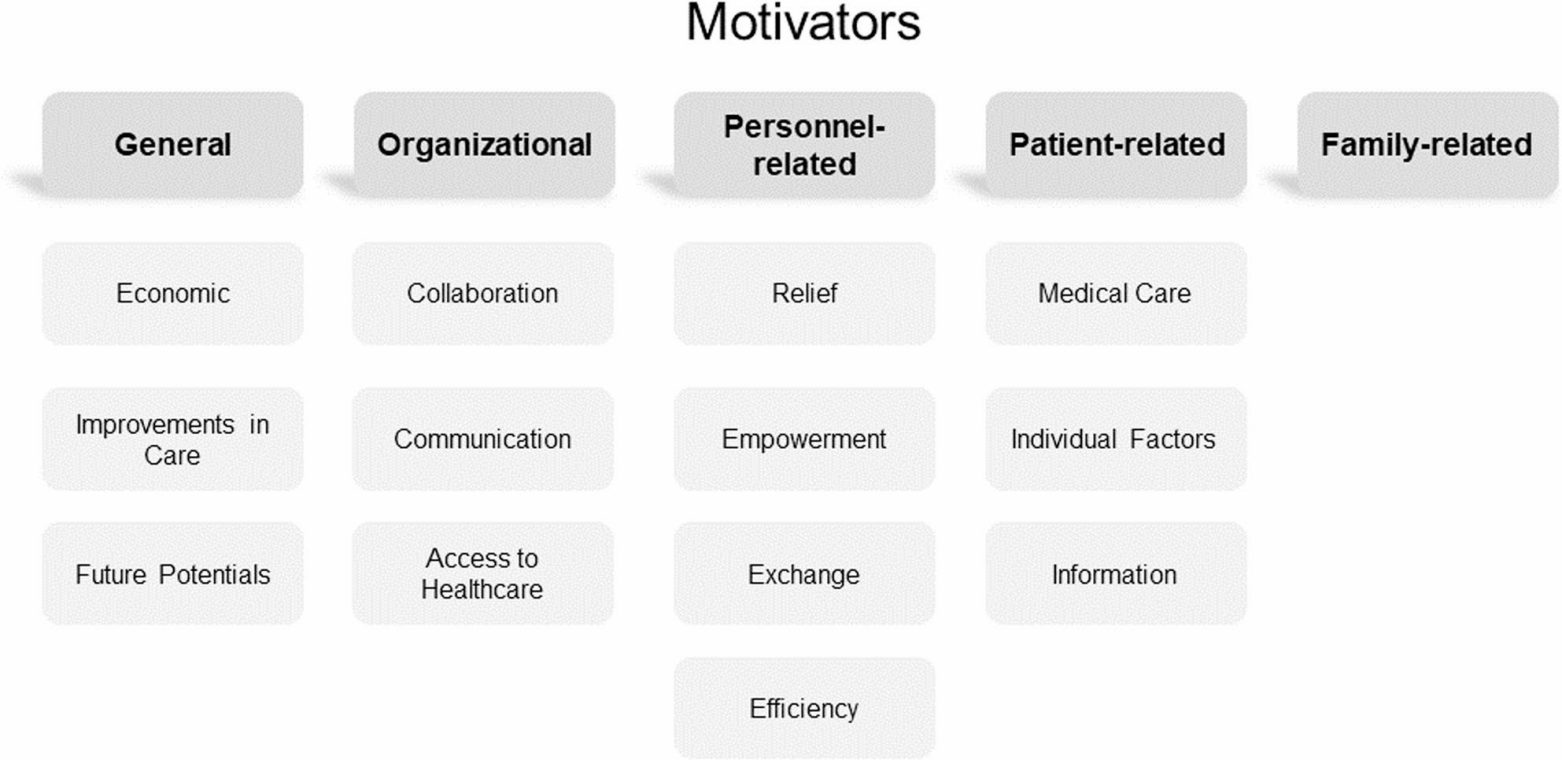
Main and subcategories of identified motivators

Taking the results of the 16 investigated studies together, 53 single aspects were identified as potential motivators of using telemedicine (see Appendix 2). These aspects were categorized into main and subcategories (see Fig. 2).

First of all, within the main category of General motivators, *economic* aspects as well as factors related to *improvements in care* and *future potentials* were categorized. *Economic* motivators were described in half of the studies, predominantly focusing on reduced overall medical costs in caregiving and an increased efficiency in care. In parts, economic advantages were described in terms of saving time as a resource and a decreased need for community paramedics. The subcategory *improvements in care* included quick access to healthcare services, an improved quality of care, the possibility to reach more homebound patients as well as the chance to tackle the lack of specialized care in remote areas. As *future potentials* the opportunities of open communication, an innovative way to deliver healthcare services and the broad applicability of providing telemedical services in different application fields (e.g., general practice, wound care, dermatology) were highlighted.

The main category of Organizational motivators integrated factors related to c*ollaboration*,* communication*, and *access to healthcare*. Within the subcategory *collaboration* different aspects were mentioned as advantages of using telemedicine in inpatient and outpatient elderly care, e.g., shared medical decision making, improvement of cooperation between healthcare professionals, or the opportunity to reduce professional isolation. Beyond that, encouraging a positive communication culture in elderly care and improving information flow between hospitals and different municipal services represented central aspects referring to the subcategory *communication*. Finally, the analysis revealed different aspects referring to *access to healthcare*, e.g., including the fast availability of medical personnel’s expertise, the security of medical care during times like pandemics, or a lower frequency of visits to specialist clinics.

The category Personnel-related motivators included the subcategories *relief*,* empowerment*,* exchange*, and *efficiency.* Concerning *relief* in the professional everyday life, the use of telemedicine in elderly care was associated with reduced working hours leading to time savings, facilitation of work, and not disrupting daily routines. Related to the subcategory *empowerment*, in particular a greater involvement of care personnel, increasing employees’ abilities to receive supervision, and the full recognition of specific skills (highlighting the care personnel’s role) was important. Regarding the subcategory *exchange*, e.g., the advantages of more informed decisions, increased knowledge transfer, better team cohesion, and interdisciplinary collaboration were described as relevant motivators to use telemedical applications. Finally, the increase in overall and individual productivity, higher performance and quality goals, and the participation in technological progress represented relevant personnel-related motivators summarized in the subcategory *efficiency.*

In the main category of Patient-related motivators, the subcategories *medical care*, *individual factors*, and *information* were relevant. Thereby, the subcategory *medical care* summarized aspects such as fewer transfers, hospitalizations, and less stress, an increased safety for patients, and therapeutic benefits. As *individual factors*, increased opportunities for the elderly to remain longer at home, saving the patients’ time, emotional support for the residents, and the inclusion of the frailest patients (e.g., cognitive impairments, disabilities) represented motivators to use telemedical innovations. Finally, within the subcategory *information*, a promoted rapport between inpatient and outpatient elderly care and patients, answering patient’s questions adequately, participation in technological progress, and increasing the patients’ knowledge represented relevant motivational factors.

Finally, the systematic review also revealed overall Family-related motivators which were occasionally mentioned. Here, a promoted rapport between inpatient and outpatient elderly care and patients’ family members, relieving the families, a greater involvement of families, and a more trusting relationship with staff were described as beneficial factors of using telemedicine in elderly care.

### Barriers of telemedicine use

**Fig. 3:**
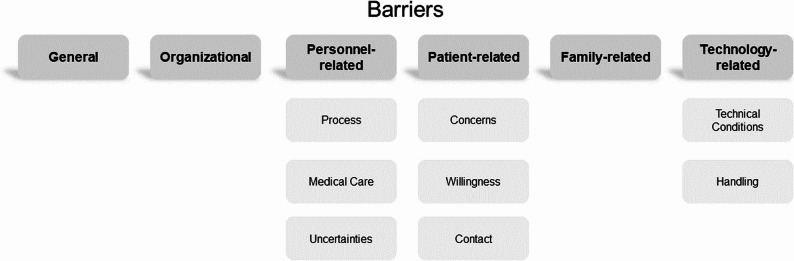
Main and subcategories of identified barriers

Taking the results of the 16 investigated studies together, 40 single aspects were identified as potential barriers of using telemedicine (see Appendix 3). These aspects were also categorized into main and subcategories (see Fig. 3).

Starting with General barriers of using telemedicine in elderly care, different aspects were described as relevant obstacles for adoption, i.e., increased costs as an economic issue, moral, ethical, and legal issues, difficulties in providing the same level of personalized care with health technology, not being able to fully replace in-person physicians’ visits, and overall skepticism towards new technologies in care.

In terms of Organizational barriers, it was identified that a dedicated room for teleconsultations is necessary to ensure patient confidentiality. Yet, this setting is not appropriate for bedridden or dependent patients. Further relevant barriers include difficulties in involving general practitioners in telemedical approaches, a low willingness of general practitioners to use telemedicine, and trust issues between medical and care personnel during telemedical consultations.

Personnel-related barriers were of major relevance as many barriers were identified in the subcategories *process*,* medical care*, and *uncertainties*. Starting with the subcategory *process*, uncertain responsibilities, difficulties to cope with changes, feelings of intrusion, and, in particular, the aspect that teleconsultations are more time-consuming (e.g., being present during the consultation, preparation, documentation, coordination) represented relevant barriers of using telemedical innovations. Related to *medical care* the studies revealed difficulties to obtain an overall impression, the assessment of a patient’s health status and condition through a screen, and language differences affecting the quality of consultations (between foreign care personnel and the local medical personnel) were identified as relevant negative factors. The subcategory *uncertainties* summarized barriers related to mistrust of new technology, increased ambiguity related to when and how to monitor and respond to patient and caregiver communication, concerns about being able to build a trusting relationship with clients, and limiting the members’ autonomy to control decisions and actions.

Patient-related barriers were also frequently highlighted and categorized into the subcategories *concerns*,* willingness*, and *contact*. First, *concerns* were identified related to an inconvenience to patients, to the idea that patients and care personnel might overuse the technology (e.g., measuring vital signs more often than needed) which leads to unnecessary distress, and concerns about the introduction of a two-tiered medicine. In addition, some barriers were identified related to the subcategory *willingness*, e.g., the inappropriateness of telemedicine for residents with cognitive impairment, unfamiliarity with the technology, concerns about the residents’ ability to readily adopt telemedicine as a form of technology-enabled care, and low willingness of residents to receive telemedical care as perceived by care personnel. Finally, losing personal contact and social presence as well as necessary support of patients from relatives or care personnel to set up the telemedical setting, were identified as relevant barriers of using telemedicine (within the subcategory *contact*).

Only one aspect was identified as Family-related barrier, related to difficulties in obtaining family consent in several cases.

As an additional category (compared to the motivators), the systematic review identified Technology-related barriers in terms of *technical conditions* and *handling*. In more detail, barriers regarding *technical conditions* referred to the technology itself, technical difficulties, and necessary technical infrastructure, concerns of a poor hardware quality and existing sensory deficits. Considering the *handling* of technology, poor security, confidentiality and reliability represented relevant concerns. Further, difficult technology handling was concerned leading to lengthened consultation durations, delays or problems with transmissions, while concerns regarding a low visual and audio quality hindering the flow of information delivery were also identified.

### Additional results

The systematic review revealed that the analyzed studies did not conduct systematic investigations of user-related factors influencing the acceptance of using telemedicine in inpatient and outpatient elderly care. In parts, some of the studies (i.e., 53, 54, 58, 59) identified demographic characteristics to be relevant for the acceptance of telemedicine focusing on age, gender, as well as the duration of technology usage or the location of care settings. These were predominantly quantitative studies leading to partly contradicting results. In addition, other studies (i.e., 48, 52, 59, 60) identified and analyzed relevant factors on a descriptive level taking contextual factors and the perspectives of different stakeholders into account (e.g., patients and professionals).

## Discussion

The included studies revealed predominantly positive attitudes of care personnel towards telemedical applications in inpatient and outpatient elderly care, but also showed some critical views and ambivalent opinions. As part of that, a range of motivators and barriers to the use of telemedicine was expressed, with more motivators than barriers revealed across all included studies. These motivators and barriers were categorized into general, organizational, personnel-related, patient-related, family-related, and technology-related aspects. While these categories offer a comprehensive overview, their relative influence on acceptance appears to differ. It is evident that organizational factors, including leadership support, integration into existing workflows, and the availability of resources, have been identified as being of critical importance for the successful and sustainable implementation of new systems and processes. Personnel-related aspects, particularly the perceived clinical benefit, professional competence, and self-efficacy, frequently mediate the translation of organizational readiness into practice. In contrast, patient- and family-related factors, while relevant in shaping day-to-day use, often exert their influence only when supportive organizational structures and motivated personnel are in place.

Telehealth projects in inpatient and outpatient elderly care are being increasingly implemented [17, 61], relying substantially on the care personnel to be successfully applied, which highlights the importance of acceptance in this occupational group. Based on the identified motivators and barriers, several requirements and strategies can be derived for the successful and sustainable implementation of telemedical applications in the elderly care sector. Involvement at the micro, meso, and macro levels of the healthcare system is necessary to ensure a sustainable delivery of telemedicine. Furthermore, these categories do not act in isolation, but rather interact in ways that shape overall acceptance [62]. For instance, the organizational culture may either serve to reinforce or attenuate patient-related barriers, such as limited digital literacy or reluctance towards tele-medical encounters. In a similar manner, personnel engagement exerts a significant influence on the effective implementation of organizational measures, such as the establishment of standardized workflows, in daily practice. Notwithstanding the existence of structural conditions, acceptance may remain constrained if healthcare professionals perceive telemedicine to be incongruent with their professional values or as an additional burden. Conversely, robust individual motivation and positive experiences have been shown to offer partial compensation for structural or technological challenges. This finding underscores the dynamic interplay between categories.

Study participants highlighted key challenges regarding telemedicine, particularly ethical and legal issues, including informed consent, patient privacy, data protection and security, quality of care, and the patient-healthcare professional relationship. From a nursing perspective, these concerns overlap with broader ethical debates about the quality of care, dignity, and patient comfort. For example, nursing research has emphasized the importance of ethical sensitivity and the relational responsibility of nurses in ensuring patient comfort during complex or painful procedures [63]. Integrating these perspectives can improve our understanding of how the ethical and relational aspects of nursing practice influence the acceptance and sustainability of telemedicine. Additionally, questions about costs, reimbursement, and insurance coverage were expressed as unresolved [26, 64, 65]. Therefore, standardizing and defining regulatory processes for telemedicine is essential. While some ethical frameworks and guidelines exist [66, 67], evidence on their practical implementation is still lacking [26]. Clear ethical and legal guidelines are necessary to support care personnel’s actions in telemedicine and ensure its sustainable implementation.

Organizational and technological factors are crucial as well. To address organizational challenges, standardized workflows involving all healthcare professionals, additional administrative tasks, including time spent on them, and private spaces for telemedical sessions need to be introduced [52, 68]. Addressing technology-related barriers requires sustainable training and education for care personnel and other healthcare professionals in using telemedical applications like videoconferencing [68, 69]. Ideally, this is already part of the regular curriculum of healthcare professionals [70]. Training should be provided on a constant basis, and if needed, refresher training for personnel with low uptake or special need for assistance could be implemented. If questions and problems arise during the regular use, a support service should be available. Since the telemedical hardware needs to be user-friendly and fit-for-purpose, an automated feedback system within the telemedical application should be implemented, allowing the technology to be iteratively improved and adapted to the user’s needs [68, 71].

Personnel- and patient-related factors were found to be relevant as well, with included studies highlighting challenges regarding the establishment of a trusting relationship and virtual health assessment. These identified challenges are in line with previous literature and studies which also identified trust to be a relevant facet and condition within the field of telemedical interactions [e.g., 72, 73]. These findings also resonate with nursing-specific literature that emphasizes the centrality of relational care and the challenges of sustaining caring relationships in digital contexts. Recent qualitative studies, for example, highlight how nurses experience tensions between efficiency and the relational dimensions of care when using telecare, underscoring implications for both patient well-being and professional identity [30].

Here, information and communication strategies should consider and emphasize that telemedical applications are used as a complement to traditional face-to-face medicine and not as a complete substitute [64]. Moreover, if a healthcare professional is uncertain about a patient’s health status during a video consultation, it is necessary to initiate contact with other professionals. Physician assistants can also provide support at this point.

Although predictors of acceptance were not systematically investigated in the included studies, potential factors, such as age, gender, care setting, and prior technology experience, emerged, consistent with broader nursing and healthcare research [74, 75]. Literature further highlights determinants such as performance expectancy, social influence, and self-efficacy as well as prior experiences with telemedicine, particularly satisfaction with and trust in the services [74–76]. These aspects need to be considered carefully in the implementation of telemedicine in order to foster a long-term integration. To further contextualize these findings, the Consolidated Framework for Implementation Research (CFIR) [e.g., 77, 78] provides a useful lens for understanding the relative importance and interaction of the identified factors. The application of a systematic approach, whereby motivators and barriers are mapped onto CFIR domains, illuminates the pivotal influence of two factors in shaping acceptance: the inner setting, comprising organizational culture, leadership, and resources, and the characteristics of individuals, encompassing personnel attitudes, demographic factors, and self-efficacy. The external environment, encompassing regulatory and ethical frameworks, along with intervention characteristics such as usability and adaptability of telemedical tools, also exerts significant influence, though frequently through organizational and individual-level processes. This perspective moves beyond listing factors, offering an implementation science–informed understanding of which aspects to prioritize in practice.

Given nurses’ central role in telemedicine implementation and delivery, strengthening the nursing lens is particularly important. Integrating insights specific to nursing on relational care, professional identity, and ethical responsibility [e.g., 30, 64] contextualizes our findings within broader nursing discussions. This enhances the contribution of this review to both nursing scholarship and health services research.

### Limitations

To our knowledge, this is the first systematic review to comprehensively analyze the status quo of acceptance by care personnel as well as their motivators and barriers to the use of telemedical approaches in inpatient and outpatient elderly care. Nevertheless, some limitations must be acknowledged.

First, the included publications are heterogeneous in terms of their characteristics and populations, which limits the comparability of the results. For instance, only four studies focused exclusively on care personnel [50, 55, 58, 60]; the remaining studies included other healthcare professions in addition to nurses. Besides, the inclusion of qualitative and quantitative methods made it difficult to summarize and generalize study findings. However, due to the limited number of studies in our target group, a broader inclusion of study types and populations allowed us to explore a wide range of possible motivators and barriers for acceptance of telemedicine by care personnel. Second, differences in study settings (inpatient vs. outpatient elderly care) and healthcare systems may further limit generalizability, although the (sub)categories identified were consistent across studies. Third, while the methodological quality of studies was mostly rated as moderate, a main flaw was the use of self-developed questionnaires instead of validated instruments. Moreover, definitions of acceptance varied, with only three studies basing their understanding on a respective theory [45, 60], which further limits the comparability of results. Furthermore, mostly small sample sizes may limit generalizability. Fourth, (sub)categories in the qualitative synthesis were determined by at least two authors, but subjectivity cannot be completely excluded. Fifth, papers not published in English or German were excluded, which may have led to a selection bias (language bias). In this context, relevant research from countries with advanced telemedicine use, such as Scandinavian and Asian countries, may not have been captured. Sixth, only three databases were searched, and the omission of CINAHL, which is relevant for nursing research, represents an additional limitation. Seventh, varying terms of telemedicine and acceptance might have affected the search strategy. To address this limitation, an additional hand search and cross-checking of references was carried out.

## Conclusions

The results of this systematic review indicate that the acceptance of telemedicine among care personnel is positive overall and that a range of motivators is present, highlighting the perceived potential of telemedicine to meet current challenges in inpatient and outpatient elderly care. However, some challenges are still present. In order to realize the full potential of telemedicine in elderly care, it is necessary to address the presented barriers. Without a general framework that supports and sustains the implementation of telemedical applications in inpatient and outpatient elderly care, care personnel will not be encouraged to use telemedicine. Future studies should make use of larger sample sizes and validated instruments to measure acceptance of telemedicine in order to generate robust and comparable results.

## Supplementary Information

Supplementary Material 1.

Supplementary Material 2.

Supplementary Material 3.

## Data Availability

No datasets were generated or analysed during the current study.

## Abbreviations

CFIR: Consolidated Framework for Implementation Research
NPT: Normalization Process Theory
PRISMA: Preferred Reporting Items for Systematic Reviews and Meta-Analyses
QuADS: Quality assessment with diverse studies
TAM: Technology Acceptance Model
UTAUT: Unified theory of acceptance and use of technology
