# Supplementary material for: Acceptance of telemedicine among care personnel in inpatient and outpatient elderly care: a systematic review
Source: BMC Geriatr. 2025 Nov 22;25:1010. doi: 10.1186/s12877-025-06786-9 (PMC12687553; doi:10.1186/s12877-025-06786-9)
Supplement: Supplementary file 1 — Supplementary Material 1. [file 12877_2025_6786_MOESM1_ESM.docx]

Appendix 1. Search strategy.

*Search strategy for MEDLINE (via PubMed)*

((((((((((((((((("attitude of health personnel"[MeSH Terms]) OR "perception"[MeSH Terms]) OR "accept*"[Title/Abstract]) OR "belie*"[Title/Abstract]) OR "satisfaction"[Title/Abstract]) OR "attitude*"[Title/Abstract]) OR "experience*"[Title/Abstract]) OR "view*"[Title/Abstract]) OR "opinion*"[Title/Abstract]) OR "perception*"[Title/Abstract]) OR "perspective*"[Title/Abstract]) OR "adoption"[Title/Abstract]) OR "implementation"[Title/Abstract]) OR "knowledge"[Title/Abstract]) OR "barrier*"[Title/Abstract]) OR "concern*"[Title/Abstract]) OR "motive*"[Title/Abstract])

AND

((((((((((((((((((((((((("telemedicine"[MeSH Terms]) OR "videoconferencing"[MeSH Terms]) OR "telemedic*"[Title/Abstract]) OR "tele medic*"[Title/Abstract]) OR "tele-health"[Title/Abstract]) OR "tele health"[Title/Abstract]) OR "telecare"[Title/Abstract]) OR "tele care"[Title/Abstract]) OR "telehomecare"[Title/Abstract]) OR "tele homecare"[Title/Abstract]) OR "telemonitoring"[Title/Abstract]) OR "tele monitoring"[Title/Abstract]) OR "telecommunication"[Title/Abstract]) OR "tele communication"[Title/Abstract]) OR "teleconsultation*"[Title/Abstract]) OR "tele consultation*"[Title/Abstract]) OR "telecooperation*"[Title/Abstract]) OR "tele cooperation*"[Title/Abstract]) OR "teleconferenc*"[Title/Abstract]) OR "tele conferenc*"[Title/Abstract]) OR "telenursing"[Title/Abstract]) OR "tele nursing"[Title/Abstract]) OR "remote health"[Title/Abstract]) OR "videoconferenc*"[Title/Abstract]) OR "video conferenc*"[Title/Abstract])

AND

((((((((((("nursing staff"[MeSH Terms] OR "nurses"[MeSH Terms]) OR "nursing"[MeSH Terms]) OR "nursing care"[MeSH Terms]) OR "nurs*"[Title/Abstract]) OR "staff"[Title/Abstract]) OR "carer*"[Title/Abstract]) OR "caregiver*"[Title/Abstract]) OR "health personnel"[Title/Abstract]) OR "professional*"[Title/Abstract]) OR "care personnel"[Title/Abstract])

AND

((((((((((((((((((((((((("ambulatory care"[MeSH Terms] OR "long-term care"[MeSH Terms]) OR "nursing homes"[MeSH Terms]) OR "home care services"[MeSH Terms]) OR "ambulatory care"[Title/Abstract]) OR "outpatient care"[Title/Abstract]) OR "home care"[Title/Abstract]) OR "homecare"[Title/Abstract]) OR "home nursing"[Title/Abstract]) OR "home health care"[Title/Abstract]) OR "home healthcare"[Title/Abstract]) OR "non-residential care"[Title/Abstract]) OR "inpatient care"[Title/Abstract]) OR "nursing home*"[Title/Abstract]) OR "care home*"[Title/Abstract]) OR "care facilit*"[Title/Abstract]) OR "longterm care"[Title/Abstract]) OR "long term care"[Title/Abstract]) OR "elderly home*"[Title/Abstract]) OR "residential facilit*"[Title/Abstract]) OR "home* for the aged"[Title/Abstract]) OR "residential aged care facilit*"[Title/Abstract]) OR "nursing service*"[Title/Abstract]) OR "residential care"[Title/Abstract]) OR "care service*"[Title/Abstract]) OR "nursing facilit*"[Title/Abstract])

Filter: English, German

Search strategy was adjusted for PsycInfo and Embase including similar search terms and restrictions.
