# Supplementary material for: Acceptance of telemedicine among care personnel in inpatient and outpatient elderly care: a systematic review
Source: BMC Geriatr. 2025 Nov 22;25:1010. doi: 10.1186/s12877-025-06786-9 (PMC12687553; doi:10.1186/s12877-025-06786-9)
Supplement: Supplementary file 2 — Supplementary Material 2. [file 12877_2025_6786_MOESM2_ESM.docx]

Appendix 2. Identified motivators of using telemedicine in elderly care.

| Category | Subcategory | Advantages | Studies |
| --- | --- | --- | --- |
| General | Economic | - Reduced overall medical costs of caregiving (incl. expenses for transfers) - Increase in efficiency of care - Saving time as a resource - Potential to decrease the need for community paramedics | 42, 45, 46, 54  51, 53–55  45  56 |
|  | Improvements in Care | - Quick access to (specialized) healthcare services - Improved quality of care - Possibility to help / reach more homebound patients - Tackling the lack of specialized care in remote areas | 46, 56  54, 55  46, 56  46 |
|  | Future Potentials | - Diverse application fields (e.g., general practice, wound care, dermatology) - Open communication - Innovative way of delivering healthcare | 44, 50  53  56 |
| Organizational | Collaboration | - Collaboration for shared medical decision making (and learning) - Improvement of cooperation between healthcare professionals - Opportunity to reduce professional isolation - Improvements in the structure of work through teleconsultations - Collaborators (e.g., medical personnel) were able to attend meetings more regularly | 49, 53  50, 53  49  44  43 |
|  | Communication | - Encouraging a positive communication culture in the care home - Improve information flow between hospitals and municipalities / different municipal services | 49  47 |
|  | Access to Healthcare | - Fast availability of medical personnel’s expertise in nursing homes - Security of medical care in nursing homes during times like pandemics - Lower frequency of visits to the specialist clinics - Increase the possibility of providing more flexible services | 42, 45  45  42  47 |
| Personnel-related | Relief | - Reduce working hours, time savings - No disruption of daily routine - Facilitation of work | 43, 44, 47, 50, 51, 54  53  53 |
|  | Empower-ment | - Full recognition of specific skills / emphasis of the care personnel's role - Greater involvement in care (empowerment) - Increase care personnel’s abilities to receive supervision, provide individualized services, participate in professional networks | 44, 46  44, 46, 51  47 |
|  | Exchange | - More informed decisions / increased knowledge transfer - Better team cohesion and interdisciplinary collaboration - Promoted rapport between nursing homes and staff - Staying in contact with patients | 42, 46, 51  43, 46  54  43 |
|  | Efficiency | - Increase of overall and individual productivity - Higher performance goals and quality targets - Participation in technological progress | 50  50  48 |
| Patient-related | Medical Care | - Fewer transfers, hospitalizations, and less stress - Increased safety for patients - Therapeutic benefits - Improvement of access to care for the residents - Provide compassionate care - Promotion of non-pharmacological treatments | 44–46, 53, 54  47, 49, 54  46, 48  47, 50  53  46 |
|  | Individual  Factors | - Increased opportunities for the elderly to remain longer at home - Saving patients' time (travelling takes much time and energy) - Emotional support for the residents - Inclusion of the frailest patients (e.g., with cognitive impairments, disabilities) | 46, 47  43  42  43 |
|  | Information | - Promoted rapport between nursing homes and patients - Answers to patient's questions - Participation in technological progress - Help to increase patients’ knowledge | 54  53  48  47 |
| Family-related | -- | - Promoted rapport between nursing homes and patients' family members - Relieve to the families - Greater involvement of families - More trusting relationship with staff | 54  47, 46  46  46 |
