# Supplementary material for: Acceptance of telemedicine among care personnel in inpatient and outpatient elderly care: a systematic review
Source: BMC Geriatr. 2025 Nov 22;25:1010. doi: 10.1186/s12877-025-06786-9 (PMC12687553; doi:10.1186/s12877-025-06786-9)
Supplement: Supplementary file 3 — Supplementary Material 3. [file 12877_2025_6786_MOESM3_ESM.docx]

 Appendix 3. Identified barriers of using telemedicine in elderly care.

| **Category** | **Subcategory** | **Barriers** | **Studies** |
| --- | --- | --- | --- |
| **General** | -- | - Increased costs, economic issues - Moral, ethical, and legal issues (medical liability as main risk) - Difficulty to provide same level of personalized care with health technology - Some visits more appropriate in person / no complete replacement of in-person physicians' visits - Overall skeptical attitudes towards new technologies in care | 46, 54  42, 54  51  45, 51  45 |
| **Organiza-tional** | -- | - Dedicated room for teleconsultations necessary to guarantee patients’ confidentiality, but not appropriate for bedridden or dependent patients - Difficulties involving medical personnel in telemedical approaches - Low willingness of medical personnel to use telemedicine as perceived by care personnel - Trust issues between medical and care personnel during telemedical consultations | 43, 50    46  45  42 |
| **Personnel-related** | Process | - Uncertainty about responsibility - Teleconsultations more time-consuming (e.g., being present during consultation, preparation, documentation, coordination) - Difficulty to cope with change - Feeling of intrusion - Lack of time and workforce for telemedicine development - Care personnel with a more subordinate role to medical personnel ("like assistants") | 54  50, 45, 44    46  46  45  52 |
|  | Medical Care | - Difficult to obtain overall impression / assessment of patient's health status and condition through screen - Language differences, affecting the quality of consultations (between foreign care personnel and local medical personnel) | 52, 43    42 |
|  | Uncertainties | - Mistrust of new technology, e.g., due to overall unfamiliarity, reluctance or low technical expertise - Increased ambiguity related to when and how to monitor and respond (i.e., via telehealth or phone) to patient and caregiver communication - Concerns of being able to build a trusting relationship with patients - Limiting the care personnel's autonomy in terms of controlling decisions and actions | 52, 54, 57  51  52  57 |
| **Patient-related** | Concerns | - Inconvenience to patients - Concern that patients and caregivers might overuse the technology (e.g., measure vital signs more often than needed), leading to unnecessary distress - Introduction of a two-tiered medicine | 54  51  46 |
|  | Willingness | - No suitability of telemedicine for residents with cognitive impairment, no benefits - Discomfort or unfamiliarity with technology - Patient engagement with a ‘new’ technology (expressed by professionals) - Concerns about the residents’ ability to readily adopt telemedicine as a form of technology-enabled care - Low willingness of residents to receive telemedical care as perceived by care personnel - Patients’ feeling to be undereducated to use the system | 45, 48  54  56  52    45  56 |
|  | Contact | - Losing personal contact / social presence - Patients will need support from relatives / care personnel to set up videoconferencing | 45, 52  43 |
| **Family-related** | -- | - Difficulties obtaining family consent in several cases | 46 |
| **Technology-related** | Technical Conditions | - Technology itself, technical difficulties & necessary technical infrastructure - Poor hardware quality - Existing sensory deficits | 43, 45, 50  54  50 |
|  | Handling | - Poor security, confidentiality, reliability - Difficult handling of tablets for patients and staff - Lengthened consultation duration - Delays or problems with transmissions, visual and audio quality hindering flow of information delivery | 54  48  42  42 |
